# An Electroencephalographic Investigation of the Impact of Eye Movements in a Memory Probe Task

**DOI:** 10.1111/psyp.70220

**Published:** 2025-12-26

**Authors:** Alberto Petrin, Sabrina Brigadoi, Mattia Doro, Paola Sessa, Roberto Dell'Acqua

**Affiliations:** ^1^ Department of Developmental Psychology and Socialization (DPSS) University of Padova Padova Italy; ^2^ Padova Neuroscience Center (PNC) University of Padova Padova Italy

**Keywords:** CDA, eye movements, ICA, saccades, SPCN

## Abstract

Lateral saccades represent a major source of noise and confounds, particularly for event‐related potentials (ERPs) that rely on hemispheric imbalances in neural activity elicited by lateralized stimuli during central fixation. These include lateralized ERPs such as the contralateral delay activity (CDA), which indexes visual working memory (VWM) load. Due to its relatively small amplitude and strict fixation requirement, the CDA is particularly vulnerable to contamination from eye movements, which usually cause the contaminated trial to be discarded. In this context, independent component analysis (ICA) offers an alternative to the traditional epoch rejection method, as it removes ocular artifacts without discarding entire trials. However, ICA's effectiveness may be limited if saccade‐related activity is not fully removed, or if trials in which participants directed their foveae toward a lateral target stimulus are retained, leading to bilateral representation. In the present study, we compared the efficacy of ICA and epoch rejection in preserving CDA features when participants were allowed to saccade. Participants were asked to memorize an array composed of a variable number of laterally displayed colored squares. In half of the experiment, participants had to keep their gaze at fixation, whereas they had to saccade toward the memoranda in the other half. The memory array was displayed for either 100 ms or 500 ms to examine how the post‐saccade physical availability of the memoranda influenced CDA amplitude and latency. The results showed that ICA correction preserved the quality and defining features of the CDA component as well as, or in some respects better than, epoch rejection. Notably, the post‐saccade physical availability of the memoranda affected the latency of the CDA, with shorter offset latency observed when the memoranda were exposed for 500 ms compared to 100 ms, likely reflecting post‐saccade retinotopic recoding of the memoranda.

## Abstract 2

Our findings definitively show that independent component analysis (ICA) can reliably correct ocular artifacts when estimating a lateralized ERP component, the CDA. The CDA memory load effect and signal quality were successfully preserved following ICA correction, even for long stimulus duration, although an earlier closure of the CDA was obtained in such cases. Participants can saccade toward stimuli upon their appearance during lateralized experimental tasks. This is possible, but certain precautions must be taken.

## 1 Introduction

Two types of event‐related lateralizations (ERLs) have become popular tools to investigate the processing mechanisms necessary to explore and maintain a stable representation of the visual world. One ERL is N2pc, which is typically studied in visual search and widely held to index the allocation of attention to laterally displayed task‐relevant (target) objects. N2pc manifests itself as a phasic negativity enhancement usually unfolding in a 200–300 ms time window at parieto‐occipital sites (i.e., PO7/PO8) contralateral to the visual hemifield in which the to‐be‐searched‐for target is displayed (Eimer 1996; Luck and Hillyard 1994). The other ERL is the contralateral delay activity (CDA; Vogel and Machizawa 2004; McCollough et al. 2007; also known as contralateral negative slow wave, or CNSW; Klaver et al. 1999; contralateral search activity, or CSA; Emrich et al. 2009; sustained posterior contralateral negativity, or SPCN; Jolicœur et al. 2008; Dell'Acqua et al. 2006; Eimer and Kiss 2010), which is popularly used to track the maintenance in visual working memory of visual information. CDA manifests itself as a protracted negativity enhancement unfolding after N2pc, starting approximately at 300 ms at the same parieto‐occipital sites contralateral to the memory array, which contains a variable number of to‐be‐remembered stimuli (Vogel and Machizawa 2004). Crucially, CDA amplitude exhibits a monotonic increase concomitant with the number of visual memoranda, though this relationship plateaus upon reaching working memory capacity, which is empirically established to be approximately 3–4 discrete items for elementary visual features such as chromatic stimuli or linear orientations.

The lateralized nature of these electrophysiological components indicates retinotopic hemispheric representations, meaning that visual information presented in one half of the visual field is processed predominantly in the opposite (contralateral) hemisphere in a spatially organized manner. Indeed, owing to the computational derivation of N2pc and CDA—wherein ERP activity ipsilateral to task‐relevant information is subtracted from homologous contralateral ERP activity—researchers usually implement stringent fixation protocols during stimulus presentation. Such methodological rigor ensures that foveae maintain consistent spatial orientation, thereby enabling reliable inference regarding which cerebral hemisphere subserves the retinotopical representation of a given visual stimulus at specific eccentricities (given a particular eccentricity; Doro et al. 2020; Papaioannou and Luck 2020).

Despite participants' best efforts to maintain fixation, saccadic eye movements still occur during some trials. These horizontal saccades introduce several artifacts into the EEG signal that can compromise data quality. The most well‐known of these artifacts include the spike potential, the corneo‐retinal artifact, and the lambda response. The spike potential is a brief, high‐frequency (~20–90 Hz), sharp biphasic deflection observed across the scalp. It is caused by the activation of extraocular muscles at saccade onset. Because its spectral and spatial characteristics resemble genuine gamma‐band neural activity, the spike potential can significantly confound EEG analyses if not properly removed (Keren et al. 2010; Plöchl et al. 2012). The corneo‐retinal artifact results from the physical rotation of the eyeball, which alters the corneo‐retinal dipole between the positively charged cornea and negatively charged retina. This produces large, slow‐voltage shifts in the EEG signal, with amplitude scaling according to saccade size. These deflections often peak over frontal sites but can spread to posterior regions, sometimes overlapping with spike potentials (Plöchl et al. 2012). The lambda response is a positive component over occipital electrodes, peaking approximately 80–100 ms after fixation onset. It reflects early visual cortical processing of the new image at the saccade landing point (Ries et al. 2018; Thickbroom et al. 1991). Unlike the other two artifacts, the lambda response is not directionally lateralized, and thus tends to cancel out in contralateral‐minus‐ipsilateral ERP waveforms such as the CDA. As a result, while the lambda response is typically minimized in lateralized ERP analyses, spike potentials and corneo‐retinal artifacts can still introduce distortions. To address these issues, it is common practice to exclude EEG epochs contaminated by saccades from further analysis. Specifically, any epoch in which the signal amplitude exceeds a predefined threshold is rejected, ensuring that only artifact‐free epochs contribute to ERP estimates.

Though ideal in point of preserving EEG data quality, epoch rejection comes at a great cost, namely data loss and, in some cases, participants' exclusion. Although data loss may be unproblematic with ERP components of large amplitude and duration (e.g., P3b) that can be easily detected with a reasonable number of trials, N2pc and CDA are relatively “small” ERP components often requiring a number of trials in the hundreds to be detected. Thus, the primary concern is not simply the trial loss but the exclusion of participants altogether, which entails wasted time and resources over and above the obvious reduction in statistical power.

To counteract this, experiments are extended beyond the duration that would be necessary in the absence of ocular artifacts, thereby including additional trials and preserving statistical power even after epoch rejection. Additionally, the rejection threshold is inherently dependent upon experimenter discretion and research group conventions, and can vary widely across laboratories (e.g., excessively stringent at 10 μV or overly permissive at 60 μV), potentially introducing methodological inconsistencies that impact the resultant data (Dell'Acqua et al. 2015; Drisdelle et al. 2017; Eimer and Mazza 2005; Meconi et al. 2018; Wang et al. 2019). Experimenters also frequently instruct participants to keep their eyes at fixation and blink only during task pauses, which can impose a dual‐task burden, increasing cognitive load, stress, and fatigue—factors that may paradoxically elevate the frequency of spontaneous ocular movements (Schleicher et al. 2008).

Diverging from the conventional epoch rejection approach, an alternative methodological solution is provided by independent component analysis (ICA). ICA correction separates independent components in EEG data, such as ocular movements from neural activity, based on their stable scalp distributions. The effectiveness of ICA is based on the assumption that it divides source signals that are not only uncorrelated but are also statistically independent, which means that the value of one variable provides absolutely no information about the value of the other (Hyvärinen and Oja 2000; Stone 2002). Researchers then exclude artifact components and recombine the remaining data, preserving a large portion of the epochs that would otherwise be rejected. This approach addresses most of the drawbacks associated with epoch rejection.

However, ICA is not immune to limitations. Depending on the algorithm used, there can be variability in how underlying sources are separated, which may lead to neural signals being mistakenly included in the artifact components (Pontifex et al. 2017). Despite these challenges, ICA remains the most widely used method for managing ocular artifacts in non‐lateralized ERP studies, with substantial evidence supporting its reliability (Delorme et al. 2007; Jung et al. 2000; Sun et al. 2005; Urigüen and Garcia‐Zapirain 2015; Zhang et al. 2024).

One question that emerges is whether ICA is suitable to correct ocular artifacts also when estimating ERLs or its use could potentially introduce bias or confounds in the data. An attempt to answer this question has been carried out by Drisdelle et al. (2017) by comparing epoch rejection and ICA in a visual search task with multiple‐frame design employing N2pc and SPCN. The paradigm involved six consecutive visual search frames separated by intervals of 900 ms ± 100 ms, after which participants reported the number of frames containing a target. The experiment consisted of two experimental blocks. In one block, participants were instructed to maintain fixation on a cross at the center of the screen; in the other block, subjects were instructed to direct their gaze toward salient items and return eye gaze to the fixation cross between each frame. The study aimed to evaluate the robustness and reliability of a preprocessing pipeline based on ICA to deal with ocular artifacts in comparison to a stricter pipeline employing the epoch rejection method.

Using these preprocessing methods, Drisdelle et al. (2017) found that ICA correction preserved over 90% of epochs compared to the epoch rejection method and did not compromise the detection of N2pc and SPCN effects. However, their study did not examine whether a key feature of SPCN—the memory load effect—was altered by ocular artifacts and their correction with ICA. Furthermore, the memory array exposure in their paradigm was very brief (200 ms), which may have led participants to suppress saccades after realizing that eye movements yielded minimal additional information, inherently reducing the actual number of potential saccades in the dataset. Additionally, the authors acknowledged that their findings may not generalize to paradigms with longer stimulus presentations, where visual information can still be acquired after an eye movement, potentially resulting in the recoding of stimulus representations across both cerebral hemispheres, rather than maintaining the lateralized processing critical for SPCN/CDA measurement.

To address these issues, we adopted a procedure similar to that used by Drisdelle et al. (2017) but with two critical modifications. First, we used a memory‐probe task with three different set sizes, which allowed us to test the memory load effect. The task was divided into two blocks (eye control conditions): in one block, participants followed traditional instructions and kept their eyes fixed at the center of the screen, avoiding blinks and saccades during stimulus presentation (central fixation condition), while in the other block, participants were instructed to saccade toward the lateral stimuli (lateral saccade condition). Second, we acquired two groups of participants: in one group the memory array was presented for only 100 ms while in the other group, it was presented for 500 ms.

This methodological design serves multiple critical purposes for the scientific community. First, this design allowed us indeed to evaluate the effectiveness of ICA in the lateral saccade condition and whether it could produce CDA estimates comparable to those obtained via epoch rejection in the central fixation condition. Furthermore, the short and long memory array exposure conditions enabled us to assess the impact of allowing participants to directly look at lateralized stimuli, thus shifting the focal eye center, and to evaluate the potential introduction of confounding variables related to saccadic movements on the CDA amplitude. Understanding the interaction between stimulus duration and artifact correction methodologies is of paramount importance for researchers, as it directly informs critical experimental design decisions regarding optimal stimulus presentation times when using either ICA or traditional artifact rejection approaches. Since we expected ICA to effectively remove saccade‐related artifacts, we hypothesized that CDA estimates would not differ significantly between the epoch rejection approach used in the central fixation condition (the gold standard) and the ICA correction applied in the lateral saccade condition. Similarly, we anticipated no significant differences in CDA estimates between the central fixation and lateral saccade conditions after ICA correction. Confirming this pattern would suggest that ICA can selectively remove artifactual activity while preserving brain signals, regardless of the proportion of contaminated trials. Additionally, we predicted an anticipated CDA offset latency for the 500 ms memory array exposure compared to the 100 ms exposure, reflecting a convergence toward a bilateral cortical representation, and thus absence of lateralization in EEG activity, once the foveae reach the lateral targets. As a secondary prediction, we expected to observe a delayed onset latency of the CDA in the fixation condition compared to the saccade condition, with the delay that could be ascribed to the additional task of maintaining fixation, which could affect the mechanisms and timing of attention allocation (Brisson and Jolicœur 2007). In brief, the experimental design enabled us to evaluate not only the effectiveness of ICA in the lateral saccade condition and whether it can produce CDA estimates comparable to those obtained via conventional epoch rejection in the central fixation condition, but also addresses a fundamental methodological question regarding stimulus duration.

To ensure a solid conclusion we conducted several control analyses. These included determining whether ICA could reduce the number of rejected epochs when compared to the epoch rejection method and whether it effectively minimized the risk of introducing confounding variables related to lateral saccades. We predicted that ICA correction would reduce the number of rejected epochs while adequately handling potential saccades' confounding variables. Additionally, we compared data quality between the two preprocessing pipelines using a recently developed metric called the “standardized measurement error” (SME; Luck et al. 2021). We also assessed whether the memory load effect was still present in the CDA component when using only ICA‐corrected epochs that were previously contaminated by ocular artifacts. We predicted an improved data quality of the CDA components after ICA correction due to the increased number of epochs available and the preservation of the memory load effect also when CDA was obtained using only ICA‐corrected epochs that were previously contaminated by ocular artifacts. Finally, we tested whether a lateralized component known as the saccade contralateral negativity (SCN; Drisdelle et al. 2017), which is related to saccade execution, was present in the contralateral‐minus‐ipsilateral difference wave after ICA correction and whether it could affect the estimation of the CDA component.

## 2 Method

### 2.1 Participants

The sample size was determined to achieve a statistical power of 0.8 for detecting memory load effects on CDA amplitude. Given the complexity of the main statistical test—a four‐way mixed‐design repeated‐measures ANOVA—we conducted a Monte Carlo simulation power analysis (Gambarota and Altoè 2024) based on the means and standard deviations of CDA amplitudes obtained from a prior pilot study. Using an alpha value of 0.05, eighteen participants were required to reach this level of power in each of two between‐subject conditions (see below).

Forty‐two students at the University of Padova (11 men, mean age = 20 years, SD = 2.1) took part in the present experiment after providing written informed consent. All participants reported normal or corrected‐to‐normal vision and no history of neurological and/or psychiatric disorders. Twenty subjects performed the task with the memory array presented for 100 ms, and twenty‐two subjects with the memory array presented for 500 ms. Six subjects were excluded from the analysis (two from the former group and four from the latter group): four participants were excluded because they had fewer than 50% of their trials remaining after artifact rejection, which involved removing trials with a high likelihood of containing blinks and saccades in the fixation condition, and blinks only in the saccade condition (see Section 2.3 for more details); one participant was removed because of technical problems during data recording; and one was excluded because they reported taking drugs for a psychiatric disorder only after finishing the task. All the other participants reported normal or corrected‐to‐normal vision and no history of neurological and/or psychiatric disorders. Thus, thirty‐six subjects were kept for the analysis: eighteen for the group with 100 ms memory array exposure time (age: *M* = 20, SD = 1.7; 5 males; 1 left‐handed) and eighteen for the group with 500 ms memory array exposure time (age *M* = 20.1, SD = 2.3; 4 males; 3 left‐handed). The experimental protocol was vetted by the local Ethics Committee (Protocol #4729). The data and scripts are available at https://osf.io/w5jqk/.

### 2.2 Stimuli and Procedure

**FIGURE 1:**
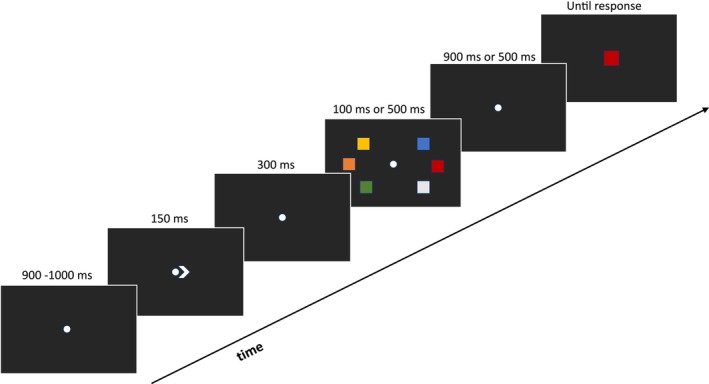
Schematic illustration of the sequence of events in one trial of the memory‐probe task. The stimuli are just approximately to scale with the stimuli displayed on the computer monitor.

The stimuli were generated with E‐Prime 2 software (Psychology Software Tools Inc.) and displayed on the dark gray (RGB: 40, 40, 40) background of a 24″ CRT monitor with a refresh rate of 60 Hz at a distance of about 60 cm. An example of the stimuli and a schematic illustration of the sequence of events is reported in Figure 1.

Each trial started with a central fixation cross, which was replaced with a 0.8 × 0.8° white (RGB: 220, 220, 220) fixation dot when participants pressed the spacebar to start a trial. The white dot remained in view throughout the trial. After a 900–1000 ms interval, randomly jittered in steps of 20 ms, a white arrow cue was exposed, with equal probability, to the left/right of the fixation dot for a 150 ms interval. After 300 ms, two arrays, each composed of 2, 3, or 5 colored squares, were displayed to the left and right of the fixation dot for either 100 or 500 ms. Each square subtended 1° × 1° of visual angle and the colors were randomly chosen from a set of eleven hues: blue (RGB: 0, 0, 255), white (RGB: 255, 255, 255), orange (RGB: 255, 153, 51), purple (RGB: 128, 0, 128), green (RGB: 0, 255, 0), cyan (RGB: 0, 255, 255), black (RGB: 0, 0, 0), magenta (RGB: 255, 102, 255), yellow (RGB: 255, 255, 51), red (RGB: 255, 0, 0), and gray (RGB: 128, 128, 128). The colored squares could be displayed in random positions inside two notional rectangles of 3.5° × 7° placed symmetrically to the left/right of the fixation dot. The distance between the fixation dot and the inner side of each rectangle was 2.5°. The minimum distance between the upper left corners of two adjacent colored squares was 1.5°. A blank retention interval of 900 ms or 500 ms (for array exposure of 100 ms or 500 ms, respectively) elapsed before the presentation of a colored square that replaced the fixation dot. With equal probability, the central color square could be one of the colors in the cued memory array or a different color. Participants had to memorize the colors displayed in the cued side of the memory array and press one of two keys (i.e., the keys “A” or “L” of the computer keyboard, counterbalanced across participants) to indicate whether the probe color was one of the colors in the memory array or a different color.

The experiment was composed of 696 trials, equally divided into two conditions. In the central fixation condition, participants were instructed to maintain gaze at fixation throughout each trial. In the other lateral saccade condition, participants were instructed to direct their gaze toward one of the to‐be‐memorized squares upon memory array onset. The order of administration of the fixation and saccade conditions was counterbalanced across participants. Participants were invited to avoid eye‐blinks before providing a response. Each condition included 116 trials for each memory array set size. Participants were exposed to 18 practice trials before the actual experiment, which took about 1 h. Half of the participants completed the experiment with memory arrays exposed for 100 ms and the other half with memory arrays exposed for 500 ms. These two memory array exposure conditions were included to evaluate the impact of giving participants sufficient time to foveate the lateralized stimuli on CDA amplitude and latency measures.

### 2.3 EEG Preprocessing (Epoch Rejection Versus ICA)

**FIGURE 2:**
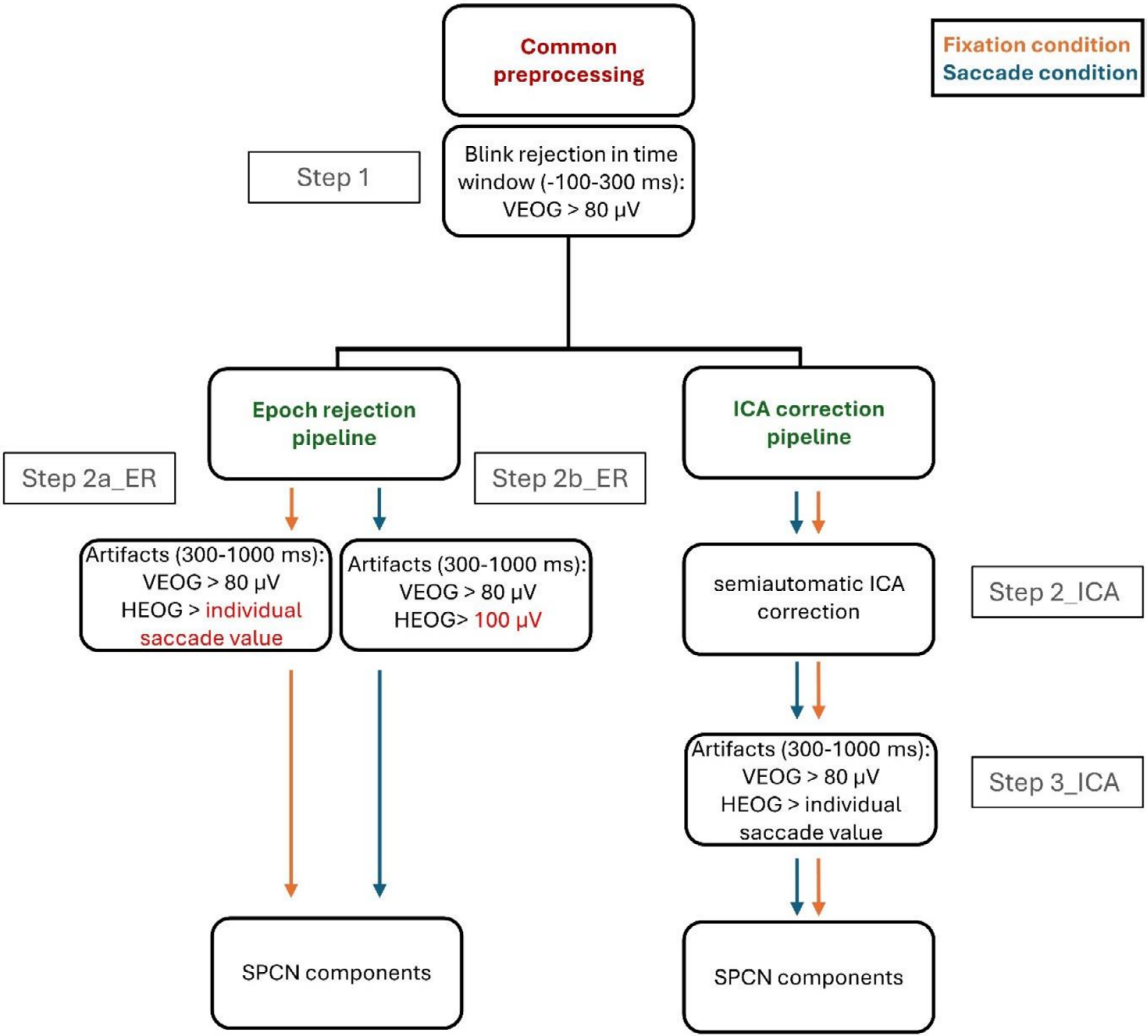
Overview of the EEG preprocessing pipelines. After a common preprocessing stage (Step 1), including blink rejection in the −100 to 300 ms window, EEG data were processed using two alternative artifact correction approaches: an epoch rejection pipeline (Steps 2a_ER and 2b_ER) and an ICA correction pipeline (Step 2_ICA). In the epoch rejection pipeline, artifacts in the 300–1000 ms window were rejected based on VEOG and HEOG thresholds, which varied by condition. For the fixation condition, HEOG artifacts were defined as exceeding an individualized saccade value; for the saccade condition, a fixed 100 μV HEOG threshold was used. In the ICA pipeline, ocular components were identified with the Eye‐Catch plugin and removed after visual inspection. After ICA correction, the same post‐correction artifact thresholds were applied (Step 3_ICA).

EEG activity was recorded from 64 active electrodes placed on an elastic Acti‐Cap according to the 10/20 International System, referenced to the left earlobe. EEG activity was re‐referenced offline to the average of the left and right earlobes. Horizontal EOG (HEOG) activity was recorded as the voltage difference between electrodes placed at the external canthi of the left and right eye. Vertical EOG (VEOG) activity was recorded as the voltage difference between an electrode placed below the left eye and Fp1. Electrode impedance was kept below 10 KΩ. EEG, HEOG, and VEOG activities were amplified and digitized at a sampling rate of 500 Hz and resampled offline at 250 Hz. EEG activity was band‐pass filtered at 0.01–30 Hz. Electrodes with values exceeding the mean activity value by more than three standard deviations were interpolated with EEG values recorded from adjacent electrodes. EEG activity was segmented into 1100 ms epochs, starting 100 ms before the onset of the memory array. EEG epochs were baseline‐corrected based on the mean activity during the −100–0 ms pre‐stimulus period. EEG epochs associated with incorrect responses or with artifacts other than eye movements (i.e., EEG activity exceeding ±100 μV within 1000 ms following the memory array onset) and/or with blinks occurring during memory array exposure (i.e., VEOG activity exceeding ±80 μV in a −100–300 interval relative to memory array onset—step 1 in Figure 2) were excluded from analysis. After these previously described common steps, the two pipelines (epoch rejection and ICA) followed different paths, as described in the flowchart of Figure 2 and in the following paragraphs. Note that EEG preprocessing did not differ between the 100 ms and 500 ms memory array exposure conditions.

Participants with more than 50% of trials rejected due to artifacts or less than 40 lateralized trials (contralateral/ipsilateral to the target) per condition were also expunged from analysis. The 50% criterion is commonly used in the literature, including by Drisdelle et al. (2017), whose study closely aligns with ours in terms of design and goals. Given this precedent, we adopted the same threshold. However, unlike Drisdelle and colleagues, we also excluded participants if the 50% threshold was exceeded in the lateral saccade condition (excluding saccadic artifacts). This 50% threshold provided a balance between data quality and retention: it was lenient enough for a paradigm in which participants were explicitly instructed to perform eye movements while still ensuring a sufficient number of usable trials for analysis. We also used the same criterion when evaluating the amount of saccade‐related activity in both the fixation and saccade conditions, based on individual saccade values.

Contralateral‐minus‐ipsilateral ERPs were generated by averaging EEG epochs recorded at PO8 on trials with cues displayed to the left of fixation and EEG epochs recorded at PO7 on trials with cues displayed to the right of fixation. Ipsilateral ERPs were generated using the opposite electrode‐side pairings. The CDA was computed by subtracting the ipsilateral activity from the contralateral activity.

#### 2.3.1 Epoch Rejection Pipeline

EEG epochs contaminated by blinks exceeding ±80 μV within the 300–1000 interval relative to memory array onset were excluded from the analysis (step 2a_ER in Figure 2). This VEOG criterion is largely used in the literature and reflects the average electrical activity of a blink (Brisson and Jolicœur 2007; Drisdelle et al. 2017; Eimer and Mazza 2005; Meconi et al. 2018).

Literature lacks a standardized criterion for HEOG artifact rejection primarily because HEOG amplitude depends on saccade amplitude. However, even in studies with similar experimental designs and comparable stimulus eccentricities, criteria vary widely, as thresholds are often selected based on the number of epochs retained for subsequent analyses (Drisdelle et al. 2017). Given the variability in HEOG amplitude (10–60 μV) due to electrode placement and individual differences, here a customized saccade threshold for each participant was computed. This was possible since half of the experimental data were heavily contaminated by saccades (during lateral saccade condition). To compute the customized threshold, first, all trials with values exceeding ±80 μV, likely representing blink‐related activity, were discarded. Then, for each remaining trial, the mean activity in a 300‐ms time window centered at the maximum absolute value of each trial was computed. Trials with mean amplitudes lower than 10 μV, which can be ascribed to negligible eye movements lower than 0.6° (Lins et al. 1993), were further discarded. This procedure ensured that only eye movements large enough to affect the retinal position of the lateral memory array contributed to participant‐specific saccade amplitude, while very small deflections (e.g., microsaccades during central fixation) were retained, as they were unlikely, given our stimulus eccentricity, to shift gaze onto the targets or meaningfully influence CDA lateralization. The *individual saccade value* was obtained by averaging the remaining mean activity values for each participant.

The HEOG criterion for the central fixation condition was therefore defined as a deflection exceeding ± *individual saccade value* in a 0–1000 ms interval following the memory array onset (step 2a_ER in Figure 2). Instead, for the lateral saccade condition, a more lenient threshold for the HEOG criterion (HEOG deflection exceeding ±100 μV within a 1‐s time window after memory array onset) was used (step 2b_ER in Figure 2), to avoid discarding most of the trials and with the aim of assessing the effect of lateralized ocular movements on the averaged lateralized ERPs.

#### 2.3.2 ICA Pipeline

ICA was performed on segmented data for each subject to identify and subsequently remove components representing ocular artifacts (step 2a_ICA in Figure 2). Data processing was performed using custom MATLAB code that called functions in the EEGLAB (Delorme and Makeig 2004) toolbox and the ERPLAB plugin (Lopez‐Calderon and Luck 2014) of EEGLAB. The EEGLAB runica routine, which implements the infomax (information maximization) ICA algorithm (Bell and Sejnowski 1995), was used since it is one of the most used and reliable ICA algorithms (Delorme et al. 2012; Pontifex et al. 2017). After decomposing the EEG data into 32 independent components representing distinct source signals, ocular‐related components were identified using the Eye‐Catch plugin. Eye‐Catch is specifically designed to detect eye movement–related components with high sensitivity and specificity (Bigdely‐Shamlo et al. 2013). Eye‐Catch operates by correlating the scalp map projection of each ICA component with a database of over 3452 exemplar eye activity‐related template scalp maps. The components selected by the Eye‐Catch plugin showing the characteristic features of blinks or saccades and associated with a *p* > = 0.8 of reflecting ocular activity according to IClabel (Pion‐Tonachini et al. 2019) were manually removed after visual inspection. No strict threshold was applied to differentiate between blink and saccade components based on *p*‐values alone, as both were treated as ocular artifacts requiring removal if they met the criteria above.

After ICA correction, the same EOG threshold criteria used for the fixation condition (deflection exceeding ±80 μV within the 300–1000 interval relative to memory array onset for VEOG and deflection exceeding ± *individual saccade value* within a 1‐s time window after memory array onset for HEOG) were applied (step 3_ICA in Figure 2), in order to ensure an appropriate comparison of saccade‐contaminated and ICA‐corrected data.

### 2.4 Statistical Analyses

#### 2.4.1 Characterizing Ocular Activity

We quantified the number of trials containing a saccade across eye control and memory array exposure conditions. Because no eye‐tracker was available, we relied on a two‐step procedure. First, we identified trials in which saccade‐related activity exceeded an individual saccade value threshold, calculated by retaining HEOG traces with mean amplitudes below 10 μV. This more lenient *individual saccade value* criterion maximized the detection of trials with high likelihood to truly contain a saccade (see Section 2.3.1. in the method section). Participants were excluded if they exhibited saccades in more than 50% of trials in the fixation condition or in fewer than 50% of trials in the saccade condition. Second, we visually inspected HEOG traces for participants whose saccade proportions were close to the 50% cutoff in the lateral saccade condition to confirm that saccades were indeed executed.

We also assessed whether the saccades were directed toward the cued hemifield, which suggested that participants' saccades landed on the target, at least in the 500 ms memory array exposure condition. To evaluate potential block order effects, we estimated the number of trials with saccades across conditions and conducted a two‐way analysis of variance (ANOVA) with exposure duration and eye control condition order (fixation‐first vs. saccade‐first) as between‐subjects factors.

Saccade onset and offset latency were estimated as the time point at which the HEOG signal reached 50% of its peak amplitude, following common practice in CDA latency estimation. We analyzed the onset latency using a repeated‐measures mixed ANOVA with set size and memory array exposure condition as factors, to assess whether participants initiated saccades earlier or later in specific conditions—a factor that could potentially invalidate the results. To estimate landing timing, we also identified the saccade landing time as the point of maximum amplitude (between saccade onset and offset) at which the derivative of the HEOG signal approached zero. This corresponds to the “elbow point,” where the saccade amplitude levels off, indicating target acquisition.

Finally, following Drisdelle et al. (2017), we estimated the proportion of trials retained using ICA correction versus epoch rejection. The total number of trials excluded using the epoch rejection approach in both the central fixation and lateral saccade conditions was calculated based on automatic removal of epochs with VEOG exceeding ±80 μV or HEOG exceeding ± the *individual saccade value*. These results were compared to trials excluded using the same criteria after ICA correction. For consistency, for this analysis only, the individual saccade value was applied also to the lateral saccade condition for the epoch rejection approach, despite a more lenient HEOG criterion being used in the EEG analysis.

#### 2.4.2 Comparison Between Preprocessing Pipelines

The statistical analyses were conducted using R and Rstudio (version 4.2.1). Accuracy data were analyzed with a generalized random mixed model with random intercepts. CDA mean amplitude was calculated considering a measurement window of 300–900 ms after the onset (Vogel and Machizawa 2004) of the memory array and submitted to repeated‐measures mixed ANOVAs. The onset and offset latency of the CDA were calculated using the jackknife procedure and measured the latency at 50% of the waveform peak grand‐averaged amplitude (Drisdelle et al. 2017; Kiesel et al. 2008), after applying a pass band filter of 0.01–10 Hz to eliminate high‐frequency waves within the time window of interest, which could compromise the estimation of latency scores. Both onset and offset latencies were submitted to a repeated‐measures mixed ANOVA, adjusting the *F* statistics with an appropriate correction ($\;F_{c}=F/{\left(n-1\right)}^{2}$; see Kiesel et al. 2008). The *t* statistics of the *post hoc* tests were adjusted with the appropriate formula ($t_{c}=t/\left(n-1\right)$; see Kiesel et al. 2008). To assess whether the anticipated CDA offset latency in the 500 ms compared to the 100 ms exposure condition was driven by convergence toward a bilateral cortical representation, we examined the temporal alignment between saccade landing and the onset of CDA offset (CDA decline onset). As with the estimation of saccade landing, the CDA decline onset latency was defined as the point of maximum negative amplitude (between component onset and offset) where the derivative approached zero, indicating the beginning of the return to baseline. We computed jackknife‐based latency estimates for both saccade landing time and the CDA decline onset latency (in the saccade condition only). For each condition, we calculated the temporal difference between saccade landing and the CDA decline onset. To analyze these values, we applied Smulders' formula (Smulders 2010) to obtain participant‐level pseudo‐latencies, which were then entered into a repeated‐measures mixed ANOVA.

All the assumptions of ANOVA were met and corrections for sphericity violations with the Greenhouse–Geisser epsilon were applied when appropriate (Jennings and Wood 1976). *Post hoc* tests, performed when appropriate, were corrected for multiple comparisons with the false discovery rate method of Benjamini and Hochberg (1995).

#### 2.4.3 Control Analyses

Several control analyses were conducted to further evaluate the effectiveness of ICA correction. First, we tested whether ICA retained more epochs compared to the epoch rejection approach and thus increased data quality. Second, we evaluated whether saccades could be a confounding factor in the ERP estimates, biasing the memory load effect, even after ICA correction. Third, we assessed the full effectiveness of ICA correction by comparing CDA estimates computed from only epochs contaminated by ocular artifacts with CDA estimates based on epochs corrected with ICA but previously contaminated by ocular artifacts. Finally, we investigated the presence of ocular‐related cognitive components, specifically the SCN, and their potential influence on the CDA.

##### 2.4.3.1 Data Quality (SME Analysis)

To evaluate whether incorporating artifact correction during preprocessing enhanced data quality compared to using epoch rejection alone, we computed a recently developed metric called the “standardized measurement error” (SME; Luck et al. 2021). SME values and the increase or decrease in root mean squares (RMS) SME were calculated following the guidelines provided by Zhang and colleagues (Zhang et al. 2024; Zhang and Luck 2023) on the CDA values estimated after both preprocessing pipelines.

##### 2.4.3.2 HEOG as Confound for the Memory‐Load Effect

The magnitude of saccades increases with the size of the set of objects in the to‐be remembered memory array (Kang and Woodman 2014), similarly to the memory‐load effect. Although previous research has shown that saccade activity is not correlated with individual visual working memory capacity, incomplete saccadic artifact removal by ICA could lead to residual activity at posterior electrodes, which may artificially enhance or distort the memory load effect, compromising the validity of inferences drawn from the data. To evaluate potential saccade‐related confounds in the ERP, we analyzed the HEOG signal before and after ICA correction for both central fixation and lateral saccade conditions. Significant HEOG amplitude differences between set size conditions would indicate that saccades could be a confounding factor, whereas no differences in the ICA‐corrected HEOG signal would suggest that ICA effectively mitigated saccade‐related confounds. To perform this analysis, the average HEOG signals in the window 300–900 ms after memory array onset were estimated, pre‐ and post‐ICA, excluding all trials with a deflection > 80 μV (likely related to blinks) and submitted, separately for each memory array exposure time condition, to a repeated measures ANOVA.

As further analysis to evaluate the ability of ICA to remove saccades propagated to posterior electrodes, the amount of saccade activity that ICA was able to remove in the posterior electrodes was compared with the expected volume conduction of saccades. Based on the normative values provided by Lins et al. (1993), we would expect a lateral saccade propagated voltage of approximately 1% ± 4% at Pz, and 0% ± 3% at Oz. Although propagation values for PO7 and PO8 were unavailable, we assumed they were similar to those at Pz and Oz. The amount of saccadic activity removed by ICA at posterior electrodes was quantified by subtracting the CDA amplitude in the lateral saccade condition after ICA correction (that should be ideally free of saccade activity) from the CDA amplitude obtained in the same condition pre‐processed with the epoch rejection method (where saccadic activity was preserved due to a higher HEOG threshold). This difference should represent the saccade activity propagated to posterior electrodes and, if ICA reliably eliminated saccade activity at posterior electrodes, should correspond to the volume conduction of saccades to posterior electrodes, approximately 2%–3% of the saccade activity observed at the HEOG electrodes. Consistent removal of saccade‐related activity at posterior electrodes would confirm ICA's ability to mitigate confounding variables associated with saccades in posterior electrode signals.

##### 2.4.3.3 CDA With Only Contaminated Trials

The goal of this control analysis was to assess the presence of a memory load effect and to examine its interaction with memory array exposure time conditions and with the methods of analysis (with vs. without ICA correction), when CDAs were estimated using only epochs identified as contaminated by ocular activity. Specifically, we selected epochs containing either blinks (VEOG deflection > 80 μV within 1 s after memory array onset) or saccades (HEOG deflection > lenient *individual saccade value*) and reanalyzed them both with and without ICA correction. The more lenient criterion used for the HEOG threshold was adopted to ensure the inclusion of all epochs with a high likelihood to truly containing a saccade. Individual CDAs were obtained averaging all uncorrected or ICA‐corrected contaminated epochs irrespective of the eye‐control condition, since no differences in terms of saccades between the two conditions are expected. CDA individual mean values were submitted to a repeated‐measures 2 × 3 × 2 mixed ANOVA, examining the memory array exposure time condition (between‐subjects, two levels: 100 ms and 500 ms), the set size of the memory array (within‐subject, three levels: 2, 3, and 5 items) and methods of analysis (within‐subject, two levels: with ICA correction and without ICA correction).

##### 2.4.3.4 SCN Component

For this analysis, we used the same contralateral‐minus‐ipsilateral difference wave components obtained in the Section 2.4.2. The mean amplitude in the 275–375 ms time window (as defined in Drisdelle et al. 2017) was submitted to a repeated‐measures mixed ANOVA to evaluate the presence and effects of the SCN component. This analysis allowed us to test whether a lateralized negativity consistent with the SCN was present across conditions, whether it was reduced by ICA correction, and whether it interacted with memory‐related modulations typically attributed to the CDA.

## 3 Results

### 3.1 Characterizing Ocular Activity

To verify compliance with the eye‐control instructions, we quantified saccade‐related activity in both the fixation and saccade conditions using the more lenient *individual saccade value* (Table 1).

**TABLE 1:** Percentage of trials with high likelihood of containing a saccade (mean, standard deviation, and range) in the 100 and 500 ms memory array exposure condition in each eye control condition, after applying the lenient individual saccade value (i.e., keeping in the estimation of the threshold also the trials with HEOG mean amplitude below 10 μV).

Saccades (%)			
Fixation condition		Saccade condition	
100 ms	500 ms	100 ms	500 ms
19.5% ± 16.7% (0.6%–45.4%)	10.1% ± 10.1% (1.2%–36.8%)	86.9% ± 10% (67.4%–98.5%)	79.3% ± 14.6% (50%–95.5%)

Importantly, polarity analysis of the HEOG signal indicated that in 100% of trials, saccades were directed toward the cued hemifield, confirming correct directional compliance. We further examined whether completing the fixation condition first reduced the frequency of saccades in the subsequent lateral saccade condition, despite counterbalancing condition order. When participants performed fixation first, they produced saccades in 69.9% ± 13.4% (100 ms exposure) and 68% ± 16.9% (500 ms exposure) of the saccade condition trials. When the lateral saccade condition was performed first, proportions were higher (82.4% ± 9.8% and 74.1% ± 16%, respectively). Although this trend suggested a possible order effect, the two‐way ANOVA revealed no significant differences (all *p*s > 0.07).

The mean saccade onset latency was 189.6 ms (±61.7 ms), which falls within the expected range for this type of task (Mayfrank et al. 1986), whereas the mean saccade offset latency was 660.1 ms (±168.1 ms). The ANOVA on saccade onset latency showed that it was not influenced by set size or stimulus exposure duration (*p*s > 0.08), indicating that participants tended to initiate their saccades at approximately the same time across conditions.

Finally, we compared the proportion of usable trials retained using ICA correction versus epoch rejection. Table 2 summarizes rejection rates for blinks and saccades across conditions in the window 0–1000 ms after memory array onset. For the percentage of trials rejected due to saccades, we used the more conservative *individual saccade value*.

**TABLE 2:** Percentage of epochs rejected (mean and standard deviation) in the 100 and 500 ms memory array exposure condition for blink and saccade artifacts in each experimental condition, after applying the conservative individual saccade value (i.e., removing in the estimation of the threshold the trials with HEOG mean amplitude below 10 μV).

	Blinks				Saccades			
	Fixation condition		Saccade condition		Fixation condition		Saccade condition	
	100 ms	500 ms	100 ms	500 ms	100 ms	500 ms	100 ms	500 ms
Epoch rejection	4.2% ± 6%	1.8% ± 2%	8.2% ± 8.3%	5.2% ± 6.3%	8.3% ± 8.7%	7% ± 6.9%	73.7% ± 13.6%	70% ± 16.7%
ICA correction	0.02% ± 0.07%	0.1% ± 0.4%	0% ± 0%	0.1% ± 0.3%	0.05% ± 0.2%	0.2% ± 0.2%	0.03% ± 0.07%	0.4% ± 1.1%

Overall, ICA correction dramatically reduced data loss relative to epoch rejection. Specifically, for the short memory array exposure condition, when the ICA correction was used to remove ocular movement‐related activity under fixation instructions, 99.5% of epochs originally labeled as blinks and 99.4% of epochs that would have been labeled as saccades were retained for analysis. When the ICA correction was used to remove ocular movement‐related activity in the lateral saccade condition, 100% of epochs originally labeled as blinks and 99.6% of epochs that would have been labeled as saccades were retained for analysis.

For the long memory array exposure condition, when the ICA correction was used to remove ocular movement‐related activity under fixation instructions, 92.7% of epochs originally labeled as blinks and 97.8% of epochs that would have been labeled as saccades were retained for analysis. When the ICA correction was used to remove ocular movement‐related activity in the lateral saccade condition, 97.8% of epochs originally labeled as blinks and 99.4% of epochs that would have been labeled as saccades were retained for analysis.

### 3.2 Behavioral Data

Accuracy data were analyzed with a generalized random mixed model with random intercepts examining the eye control conditions (within‐subject, two levels: central fixation and lateral saccade conditions), the time exposure of the memory array (between‐subjects, two levels: 100 ms and 500 ms), and the set size of the memory array (within‐subject, three levels: 2, 3, and 5 items) and their interactions.

**FIGURE 3:**
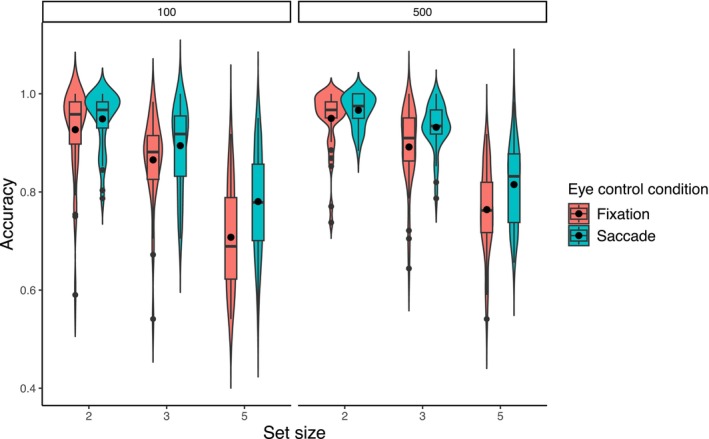
The violin boxplots represent the distribution and the quantile of subjects' accuracy at different set sizes of the memory array, divided for eye control conditions and memory array exposure condition. The dots in the boxplots represent the mean accuracy for each condition.

A graphical summary of the accuracy results is reported in Figure 3. Subjects performed significantly better at set size 2 than 3 (95.0% ± 6.5% vs. 89.6% ± 8.0%, respectively; OR = 0.46, 95% CI [0.41–0.52], *p* < 0.001) and at set size 3 than 5 (89.6% ± 8.0% vs. 77.0% ± 10.1%, respectively; OR = 0.36, 95% CI [0.33–0.40], *p* < 0.001). Furthermore, subjects were more accurate in the lateral saccade condition than the central fixation one (89.0% ± 9.9% vs. 85.1% ± 12.2%, respectively; OR = 0.68, 95% CI [0.62–0.74], *p* < 0.001). No significant differences in accuracy were found based on the memory array exposure time (OR = 0.74, 95% CI [0.51–1.06], *p* = 0.102; 85.4% ± 12.2% vs. 88.6% ± 10.0% for the 100 ms and 500 ms condition, respectively).

### 3.3 CDA Amplitude

**FIGURE 4:**
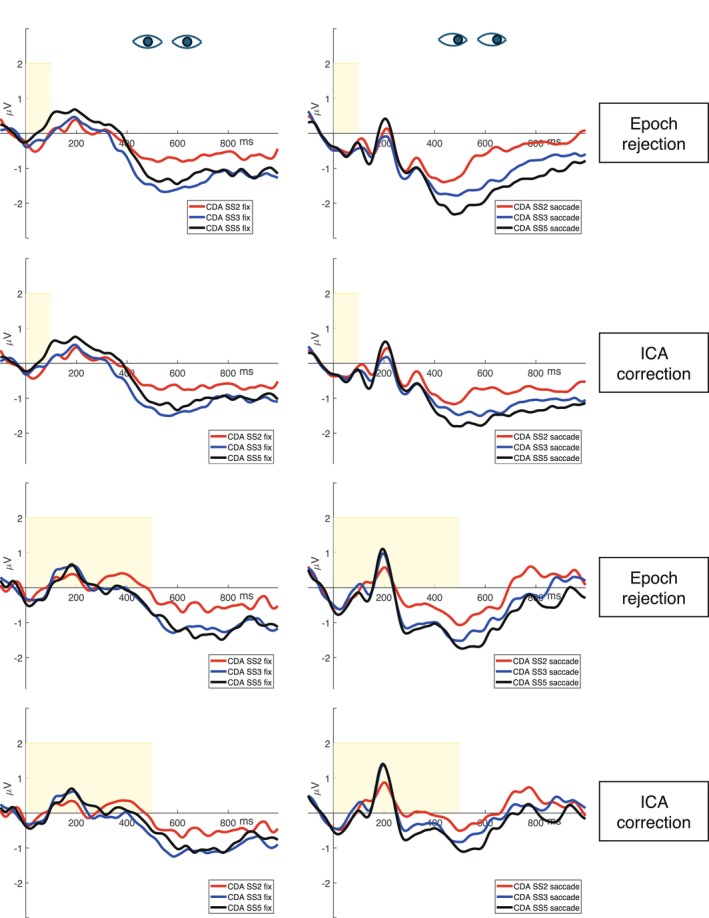
Grand‐averaged contralateral‐minus‐ipsilateral difference waves (at PO7/PO8) for each condition. The yellow areas represent the memory array exposure time (100 or 500 ms), the eyes looking straight ahead represent the central fixation condition, and the eyes looking to the right represent the lateral saccade condition. It is important to note that the eyes looking to the right are merely illustrative; in the actual experiment, saccades could be executed in both the left and right directions. To the right of the images, the preprocessing pipelines used are specified, and the different colored lines represent the set size of the memory array (2‐ red, 3—blue, or 5—black ‐items).

Figure 4 illustrates the contralateral‐minus‐ipsilateral difference waves for the two eye‐control conditions, the two preprocessing pipelines, and the two memory array exposure conditions. Mean CDA values were submitted to a 2 × 2 × 2 × 3 mixed ANOVA, examining the preprocessing pipelines (within‐subject, two levels: epoch‐rejection and ICA correction method), the eye control conditions (within‐subject, two levels: central fixation and lateral saccade conditions), the time of exposure of the memory array (between‐subjects, two levels: 100 ms and 500 ms) and the set size of the memory array (within‐subject, three levels: 2, 3, and 5 items). Table 3 summarizes the results of the 2 × 2 × 2 × 3 mixed ANOVA.

**TABLE 3:** Results of the 2 × 2 × 2 × 3 repeated‐measures mixed ANOVA on CDA amplitudes.

Effects	DFn–DFd	*F*	*p*	*η* ^2^
Exposure	1–34	2.99	0.092	0.044
Pipelines	1–34	11.26	**0.002**	0.007
Set size	2–68	21.66	**< 0.001**	0.049
Eye control	1–34	0.15	0.697	0.001
Exposure × Pipelines	1–34	4.82	**0.035**	0.003
Exposure × Set size	2–68	0.07	0.936	0.0002
Exposure × Eye control	1–34	1.40	0.244	0.011
Pipelines × Set size	2–68	7.12	**0.002**	0.001
Pipelines × Eye control	1–34	4.45	**0.042**	0.002
Set size × Eye control	2–68	2.50	0.090	0.006
Exposure × Pipelines × Set size	2–68	0.10	0.905	< 0.0001
Exposure × Pipelines × Eye control	1–34	7.94	**0.008**	0.003
Exposure × Set size × Eye control	2–68	0.24	0.785	0.001
Pipelines × Set size × Eye control	2–68	0.63	0.538	0.0001
Exposure × Pipelines × Set size × Eye control	2–68	1.80	0.174	0.0003

*Note:* Significant factors and interaction effects are reported in bold.

A significantly smaller negativity in CDA amplitude was obtained after ICA correction compared to epoch rejection (*F*(1,34) = 11.26, *p* = 0.002, *η* ^2^ = 0.007; epoch rejection method: *M* = −0.89 μV, SD = 1.25 μV; ICA correction: *M* = −0.70 μV, SD = 1.12 μV). Furthermore, a general memory load effect was found (*F*(2,68) = 21.66, *p* < 0.001, *η* ^2^ = 0.05; 2 items: *M* = −0.44 μV, SD = 1.12 μV; 3 items: *M* = −0.94 μV, SD = 1.08 μV; 5 items: *M* = −1.01 μV, SD = 1.28 μV). To characterize the memory load effect, we performed a series of FDR‐corrected (Benjamini and Hochberg 1995) post hoc tests. The CDA amplitude was significantly less negative at set size 2 than 3 (*t*(68) = 5.31, *p* < 0.001), and at set size 2 than 5 (*t*(68) = 6.02, *p* < 0.001). No difference in amplitude was observed between set size 3 and 5 (*t*(68) = 0.71, *p* = 0.50).

We observed a significant interaction between preprocessing pipeline and the time of exposure of the memory array, reflecting a larger reduction in the CDA amplitude when ICA correction was applied compared to epoch rejection when the memory array exposure was 500 ms than 100 ms (*F*(1,34) = 4.82, *p* = 0.04, *η* ^2^ = 0.003). A significant interaction also emerged between preprocessing pipeline and eye control condition, reflecting significantly smaller amplitudes in the central fixation condition compared to the lateral saccade condition when the epoch rejection method was used (*F*(1,34) = 4.45, *p* = 0.04, *η* ^2^ = 0.002). Furthermore, we found a significant interaction between preprocessing pipeline and set size of the memory array, with consistently smaller CDA amplitudes across all three set sizes when ICA correction was applied compared to the epoch rejection method (*F*(2,68) = 7.12, *p* = 0.002, *η* ^2^ = 0.001). Moreover, a three‐way interaction was observed between eye control condition, the memory array exposure condition, and preprocessing pipeline (*F*(1,34) = 7.94, *p* = 0.008, *η* ^2^ = 0.003), suggesting that the impact of preprocessing method on CDA amplitude varied depending on both the timing of the memory array and the type of eye control condition. It should be noted that the main effect of the preprocessing pipeline, as well as its interaction with the eye‐control conditions, may primarily reflect the fact that propagated saccade activity was preserved in the lateral saccade condition when epoch rejection was applied.

Post hoc tests were performed to characterize these interactions. The first test assessed whether the CDA estimated using the classical setup (epoch rejection in the central fixation condition) differed from the CDA estimated using ICA correction on artifact‐contaminated trials in the lateral saccade condition. No significant differences were observed between these two approaches (*t*(39.7) = 0.84, *p* = 0.57 for the 100 ms memory array exposure condition; and *t*(39.7) = −1.63, *p* = 0.39 for the 500 ms memory array exposure condition). The second test evaluated whether applying ICA correction to both eye control conditions would result in CDA differences. Again, no significant differences emerged in the estimated CDA between the central fixation and lateral saccade conditions following ICA correction (*t*(37) = −1.19, *p* = 0.45 for the 100 ms memory array exposure condition; and *t*(37) = −1.27, *p* = 0.43 for the 500 ms memory array exposure condition). The third test aimed to further explore the observed three‐way interaction and identify which condition was driving the effect. Specifically, we observed that the comparison between preprocessing pipelines in the lateral saccade condition when the memory array was exposed for 500 ms showed a significant decrease of the CDA mean amplitude for all the set sizes after ICA correction compared to epoch rejection (*t*(62) = 5.22, *p* < 0.001). All the other comparisons did not produce significant results (all *p*s > 0.07). No other effects or interactions between experimental factors and preprocessing pipelines were significant (all *F*s < 3.01, *p*s > 0.09, *η* ^2^s < 0.044).

Unlike the 500‐ms condition, the comparison between preprocessing pipelines in the lateral saccade condition with a 100 ms memory array exposure did not show a significant decrease in CDA mean amplitude after ICA correction relative to epoch rejection. This is likely because, as shown in Figure 6, saccade amplitude in this condition starts to decrease around 400 ms after array onset. As a result, both the HEOG signal and the saccade‐related activity propagated to PO7/PO8 electrodes reach an overall mean amplitude close to 0 μV within the CDA time window (see Figures 6e and 7).

Finally, we performed an additional analysis to determine whether the CDA memory load effect could still be observed when considering only its amplitude before return to the baseline (300–650 ms) in the long exposure condition, specifically when participants executed saccades. We found a significant memory load effect (*F*(2,34) = 4.19, *p* = 0.024, *η* ^2^ = 0.04; 2 items: *M* = −0.44 μV, SD = 1.28 μV; 3 items: *M* = −0.85 μV, SD = 1.17 μV; 5 items: *M* = −1.07 μV, SD = 1.55 μV), with significantly less negative amplitudes at set size 2 compared to set size 5 (*t*(34) = 2.85, *p* = 0.02; all other *p*s > 0.1).

### 3.4 CDA Latency

To test differences in the latency onset of the contralateral‐minus‐ipsilateral difference waves, a 4‐way mixed ANOVA (preprocessing pipeline × eye control conditions × time of exposure of the memory array × set size) was performed revealing a significant difference between eye control conditions (*F*(1,34) = 10.31, *p* = 0.003; fixation: *M* = 464 ms, SD = 47 ms; saccade: *M* = 348 ms, SD = 70 ms). No other interactions between experimental factors and preprocessing pipelines were significant (all *F*s < 3.85, *p*s > 0.06).

Then, the same 4‐way mixed ANOVA was performed to investigate differences in the latency offset of the contralateral‐minus‐ipsilateral difference waves. A significant difference was observed between eye control conditions (*F*(1,34) = 4.53, *p* < 0.04; fixation: *M* = 906 ms, SD = 67 ms; saccade: *M* = 733 ms, SD = 151 ms). No other main effects or interactions were significant (all *F*s < 2.07, *p*s > 0.16). To understand whether there was a specific condition driving this main effect, we performed a series of post hoc tests, in which we observed that in the central fixation condition when the memory array was exposed for 500 ms, the CDA offset latency was significantly delayed compared to the lateral saccade condition (*t*(34) = 2.34, *p* = 0.03). No other effects were significant (*p*s > 0.1).

To investigate whether the anticipated CDA offset latency in the long exposure condition was driven by participants' saccades landing on the memory array, we examined the temporal alignment between saccade landing and the onset of CDA offset. Estimates were calculated using only the lateral saccade condition and defined as the temporal difference between saccade landing and CDA decline onset. These estimates were analyzed with a repeated measures mixed ANOVA including preprocessing pipeline (within‐subjects, two levels: epoch rejection vs. ICA correction), memory array exposure time (between‐subjects, two levels: 100 ms vs. 500 ms), and set size (within‐subjects, three levels: 2, 3, and 5 items). The analysis revealed a single significant effect of memory array exposure time (*F*(1,34) = 4.35, *p* = 0.04, *η* ^2^ = 0.02; 100 ms: *M* = 136 ms, SD = 472 ms; 500 ms: *M* = 39 ms, SD = 172 ms), indicating that in the 500 ms condition—where CDA offset latency appeared earlier during saccades—the temporal gap between saccade landing and CDA decline onset was significantly smaller than in the 100 ms condition. No other effects were significant (*p*s > 0.4). These results suggest that only in the 500 ms condition did participants have enough time to complete the saccade and stabilize their foveae on the target.

### 3.5 Control Analyses

#### 3.5.1 SME

**FIGURE 5:**
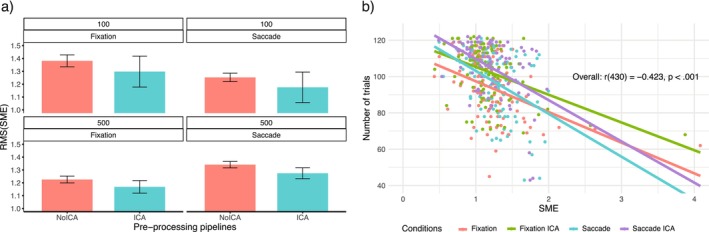
(a) (RMS(SME)) divided by preprocessing pipelines, memory array exposure conditions, and eye control conditions. Smaller RMS(SME) values indicate higher data quality. Error bars show the standard error of the RMS(SME) values; (b) correlation between SME scores and number of trials used for estimating the specific SME score.

Figure 5a shows a reduction in the RMS(SME) values after ICA correction, suggesting an improved data quality after ICA than after the epoch‐rejection method. Specifically, when the memory array was exposed for 100 ms, performing ICA correction reduced the RMS(SME) values by 6% in the central fixation condition, and by 6.2% in the lateral saccade condition. When the memory array was exposed for 500 ms, performing ICA correction reduced the RMS(SME) values by 4.7% in the central fixation condition, and by 5% in the lateral saccade condition. Overall, there was an increase in data quality of 6.1% for the 100 ms, and 4.8% for the 500 ms condition after ICA. To assess whether these increases in data quality after ICA correction were due to the higher number of trials available for each condition, a Pearson's correlation between SME values and the relative number of trials was performed, demonstrating a strong significant correlation (*r*(430) = −0.423, *p* < 0.001). The RMS(SME) scores decreased as the number of trials available increased.

#### 3.5.2 HEOG as Confound

This control analysis was devised to determine if differences in CDA amplitude related to set size (e.g., the memory load effect) could be ascribed to residual HEOG activity, and if ICA correction effectively removes such confounding variance. Individual averaged HEOG data were submitted to a 2 × 2 × 3 ANOVA, conducted separately for each memory array exposure condition, examining the ICA correction condition (within‐subject, two levels: before ICA correction and after ICA correction), the eye control conditions (within‐subject, two levels: central fixation and lateral saccade condition), and the set size of the memory array (within‐subject, three levels: 2, 3, and 5 items).

For both short and long memory array exposure conditions, HEOG amplitude values increased as the set size increased (*F*(2,34) = 66.52, *p* < 0.001, *η* ^2^ = 0.04 for 500 ms; *F*(2,34) = 49.61, *p* < 0.001, *η* ^2^ = 0.04 for 100 ms). Additionally, HEOG amplitude was significantly lower after ICA correction and in the central fixation condition compared to the lateral saccade condition. These patterns were supported by significant interactions between ICA correction and set size of the memory array (*F*(2,34) = 71.08, *p* < 0.001, *η* ^2^ = 0.03 for 500 ms; *F*(2,34) = 52.93, *p* < 0.001, *η* ^2^ = 0.03 for 100 ms) and between set size and eye control condition (*F*(2,34) = 26.34, *p* < 0.001, *η* ^2^ = 0.01 for 500 ms; *F*(2,34) = 40.76, *p* < 0.001, *η* ^2^ = 0.03 for 100 ms). A significant three‐way interaction among ICA correction, set size, and eye control condition was also observed for both exposure durations (*F*(2,34) = 33.76, *p* < 0.001, *η* ^2^ = 0.01 for 500 ms; *F*(2,34) = 45.76, *p* < 0.001, *η* ^2^ = 0.02 for 100 ms).

For the 500 ms memory array exposure condition only, additional main effects confirmed that HEOG values were reduced after ICA correction (*F*(1,17) = 43.95, *p* < 0.001, *η* ^2^ = 0.38), and in the central fixation condition compared to the lateral saccade one (*F*(1,17) = 35.67, *p* < 0.001, *η* ^2^ = 0.34). A significant interaction between ICA correction and eye‐control condition showed that, in the lateral‐saccade condition, HEOG values after ICA correction were nearly identical to those in the central‐fixation condition, in contrast to the HEOG values of both eye‐control conditions before ICA (*F*(1,17) = 38.73, *p* < 0.001, *η* ^2^ = 0.30; before ICA–fixation vs. before ICA–saccade: *t*(33.4) = −19.83, *p* < 0.001; after ICA–fixation vs. before ICA–saccade: *t*(33.5) = −1.89, *p* = 0.62), indicating that ICA effectively minimized saccadic activity.

To evaluate the effect of the ICA correction on each set size amplitude for both eye control conditions, a series of FDR‐corrected post hoc tests was performed. Before ICA correction, in the lateral saccade condition, the HEOG amplitude values were significantly more negative at set size 2 than 3 (*t*(131.6) = −12.66, *p* < 0.001 for 500 ms; *t*(131.6) = −12.24, *p* < 0.001 for 100 ms), at set size 3 than 5 (*t*(131.6) = −6.40, *p* < 0.001 for 500 ms; *t*(131.6) = −6.70, *p* < 0.001 for 100 ms), and at set size 2 than 5 (*t*(131.6) = −19.07, *p* < 0.001 for 500 ms; *t*(131.6) = −18.94, *p* < 0.001 for 100 ms). In contrast, for the central fixation condition before ICA correction this set size‐related increase in HEOG amplitude was observed only for the 500 ms condition (set size 2 vs. 5 (*t*(131.6) = −4.761, *p* < 0.001); 3 vs. 5 (*t*(131.6) = −2.59, *p* = 0.02)), although no significant difference was found between set size 2 and 3 (*t*(131.6) = −2.174, *p* = 0.06). No significant differences among set sizes were found for the 100 ms condition (all *p*s > 0.18). Crucially, after ICA correction, no significant set size effects on HEOG amplitude were found for either eye control condition or memory array exposure duration (all *p*s > 0.44). These results suggest that the observed HEOG activity scales with set size but is substantially reduced by ICA correction, particularly in the lateral saccade condition. After correction, HEOG no longer varied with set size, supporting the interpretation that the CDA memory load effect reported in the main analyses is not driven by residual eye movement artifacts.

**FIGURE 6:**
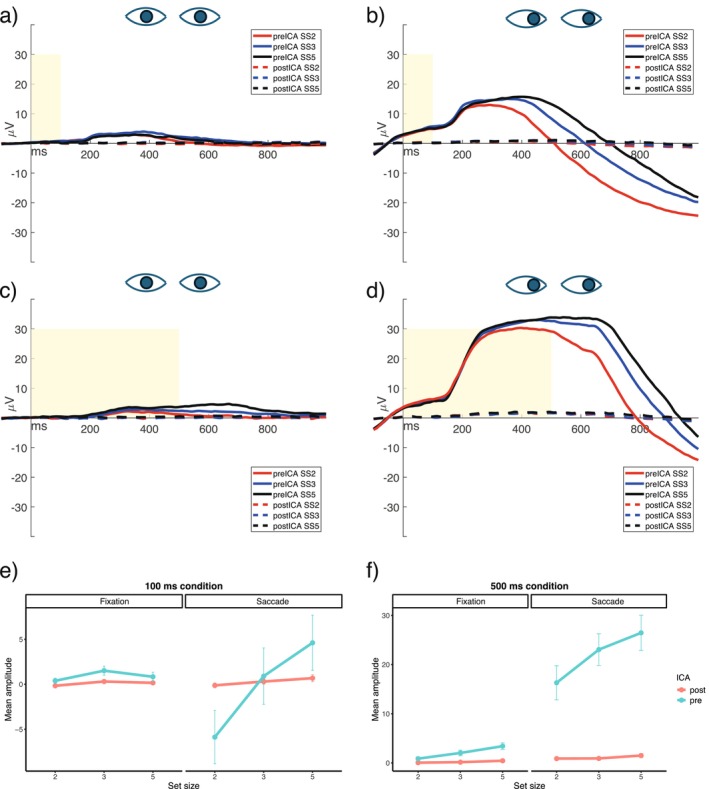
(a, b) Saccade activity in the 100 ms memory array exposure condition (the yellow areas represent the duration of the memory array, that is 100 or 500 ms) in the central fixation condition and in the lateral saccade condition (the eyes looking straight ahead represent the central fixation condition, and the eyes looking to the right represent the lateral saccade condition). The continuous lines represent the HEOG activity (before ICA correction), and the dotted lines represent the HEOG activity after ICA correction. (c, d) Saccade activity in the 500 ms memory array exposure condition in the central fixation condition to the left and in the lateral saccade condition to the right. The continuous lines represent the HEOG activity (before ICA correction), and the dotted lines represent the HEOG activity after ICA correction. (e, f) The mean amplitude in the 300–900 ms time window for the HEOG bipolar channel divided per set size. The amplitude is plotted in μV.

As illustrated in Figure 6, the different significant results between the ANOVAs in the two memory array exposure conditions may be attributed to the decrease in saccade amplitude occurring approximately 400 ms after memory array onset in the 100 ms exposure condition. This decrease resulted in an overall mean of approximately 0 μV (Figure 6e), especially when the set size values were aggregated.

**FIGURE 7:**
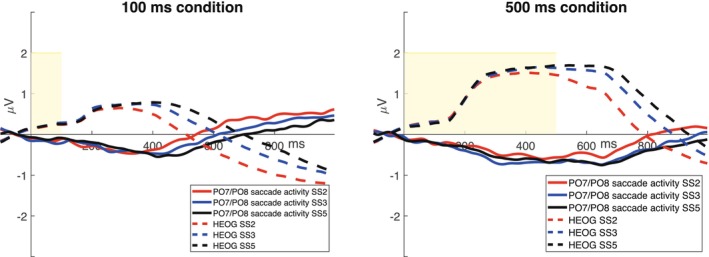
Saccade activity removed by ICA (solid lines) and propagated saccade activity (dashed lines) at PO7/PO8 scalp sites for 100 ms and 500 ms memory array exposure condition (the yellow areas represent the duration of the memory array). Note that the saccade activity has been reduced to 1/20th of its actual size for visualization purposes.

The effectiveness of ICA correction was further demonstrated by computing the saccade activity at PO7/PO8 electrodes removed by ICA and comparing it with the expected saccadic activity propagated to posterior electrodes (Figure 7). This latter was computed by taking around the 2%–3% of the maximum saccade value at HEOG electrodes across all set sizes, separately for the 100 ms memory array exposure condition (14.57 ± 1.43 μV; expected propagated value to PO7/8: ±0.30–0.44 μV), and the 500 ms memory array exposure condition (32.42 ± 1.86 μV; expected propagated value to PO7/8: ±0.65–0.97) (Figure 6b/d). The maximum value of the residual saccade activity at PO7/PO8 resulted −0.48 ± 0.04 μV for the 100 ms condition and −0.7059 ± 0.08 μV for the 500 ms condition (Figure 6). Hence, the propagated saccade activity removed by ICA at PO7/PO8 electrodes represented 2.76% ± 0.65% of the maximum value of the saccade at HEOG electrodes, in line with the normative values provided by Lins et al. (1993).

#### 3.5.3 CDA in Contaminated Trials Only

**FIGURE 8:**
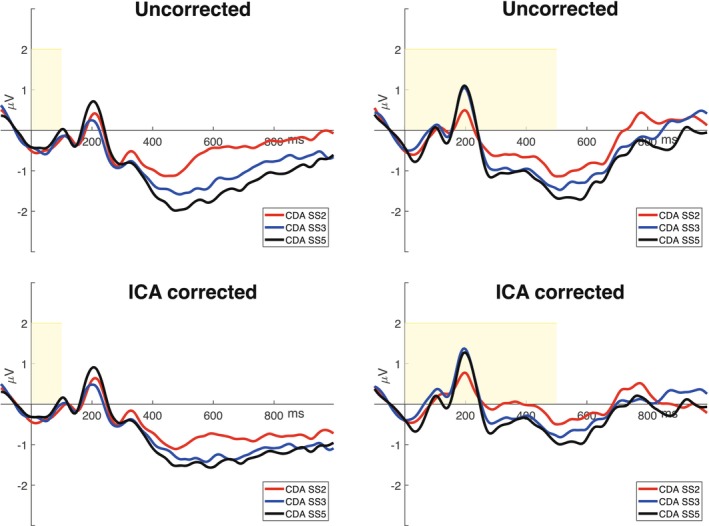
Grand‐averaged contralateral‐minus‐ipsilateral difference waves (at PO7/PO8). The yellow areas represent the memory array exposure time (100 or 500 ms), whereas the different colored lines represent the set size of the memory array (2—red, 3 ‐blue, or 5—black—items). To the right of the images, the methods of analysis used are specified.

Since this analysis focused solely on artifact‐contaminated epochs, the artifact rejection criteria used to exclude participants in prior analyses were no longer applicable. As a result, only two participants from the original dataset were excluded. Forty subjects were therefore kept for this analysis: twenty for the group with 100 ms memory array exposure time (age: *M* = 19.9, SD = 1.6; 5 males; 1 left‐handed) and twenty for the group with 500 ms memory array exposure time (age: *M* = 20, SD = 2.2; 5 males; 4 left‐handed). Figure 8 illustrates the contralateral‐minus‐ipsilateral difference waves for each condition considered.

CDA individual mean values were submitted to a 2 × 3 × 2 mixed ANOVA, examining the memory array exposure time condition, the set size of the memory array, and methods of analysis. The analysis revealed a significant difference in the CDA amplitude values between the set sizes of the memory array (*F*(2,76) = 7.77, *p* < 0.001, *η* ^2^ = 0.05; 2 items: *M* = −0.49 μV, SD = 1.31 μV; 3 items: *M* = −0.84 μV, SD = 1.25 μV; 5 items: *M* = −1.05 μV, SD = 1.34 μV), with the CDA in set size 5 and 3 being significantly more negative than in set size 2 (2 vs. 5: *t*(76) = 3.89, *p* < 0.001; 2 vs. 3: *t*(76) = 2.48, *p* = 0.02). No significant differences were obtained between amplitude at set size 3 and 5 (*p*s = 0.16). The amplitude values were also significantly more negative when ICA was not applied compared when it was, because the propagated saccade activity was maintained when the component was not corrected for artifacts (*F*(1,38) = 7.72, *p* = 0.008, *η* ^2^ = 0.009; ICA: *M* = −0.67 μV, SD = 1.25 μV; NoICA: *M* = −0.91 μV, SD = 1.37 μV).

Besides, an interaction between memory array exposure condition and method of analysis was found, indicating a significant reduction in the CDA negativity when ICA was applied only in the 500 ms exposure condition (*F*(1,38) = 9.13, *p* = 0.004, *η* ^2^ = 0.01; 500 ICA vs. 500 NoICA: *t*(38) = 4.10, *p* = 0.001), as well as an interaction between the set sizes of the memory array and the method of analysis (*F*(2,76) =10.01, *p* < 0.001, *η* ^2^ = 0.002), suggesting that failing to correct for saccade artifacts inflates the apparent CDA memory load effect. Importantly, while artifact contamination does not appear to eliminate the memory load effect, it may artificially enhance its magnitude, potentially shifting the plateau of CDA amplitude.

#### 3.5.4 SCN Amplitude

To investigate the presence and effect of the lateralized component related to saccades, initially termed SCN, we conducted a 2 × 2 × 2 × 3 repeated‐measures mixed ANOVA on the mean contralateral‐minus‐ipsilateral difference wave amplitudes within the 275–375 ms time window. The factors included in this analysis were the same as those in our main CDA analysis: preprocessing pipeline, eye control condition, memory array exposure duration, and memory array set size.

We found a significantly smaller SCN amplitude after ICA correction compared to epoch rejection (*F*(1,34) = 27.57, *p* < 0.001, *η* ^2^ = 0.01; epoch rejection: *M* = −0.49 μV, SD = 1.38 μV; ICA correction: *M* = −0.23 μV, SD = 1.20 μV). In addition, we observed a general effect of memory load (*F*(2,68) = 4.27, *p* = 0.02, *η* ^2^ = 0.01; 2 items: *M* = −0.17 μV, SD = 1.18 μV; 3 items: *M* = −0.49 μV, SD = 1.29 μV; 5 items: *M* = −0.42 μV, SD = 1.40 μV) and a greater negativity in the lateral saccade condition compared to the central fixation condition (*F*(1,34) = 18.31, *p* < 0.001, *η* ^2^ = 0.1; saccade: *M* = −0.77 μV, SD = 1.49 μV; fixation: *M* = 0.05 μV, SD = 0.90 μV).

We also found a significant interaction between preprocessing pipeline and eye control condition, indicating that ICA correction produced a larger reduction in SCN amplitude when participants performed saccades than when they maintained fixation (*F*(1,34) = 34.08, *p* < 0.001, *η* ^2^ = 0.01). These findings are consistent with the interpretation by Drisdelle et al. (2017) that the SCN reflects a distinct component specifically related to saccade execution. This issue will be further addressed in the Section 4.

## 4 General Discussion

The present study investigated the reliability of ICA in correcting ocular artifacts, particularly saccades, when estimating ERLs. Specifically, we assessed the impact of lateral saccades—and their correction via ICA—on the CDA, a lateralized ERP component indexing visual working memory load. Using a memory‐probe task with one block of trials in which participants were instructed to maintain fixation and another block in which they were allowed to saccade toward the memory array, we evaluated whether both data quality and the CDA memory‐load effect were preserved when using ICA compared to the standard epoch rejection method. In addition, we examined whether memory array exposure time modulated the efficacy of ICA by testing two participant groups, one with an array exposure of 100 ms and the other with 500 ms.

The results clearly showed that both ICA correction and epoch rejection preserved the CDA memory‐load effect across memory array exposure conditions, even when participants were allowed to saccade to the lateral stimuli. Importantly, the memory‐load effect remained robust in the CDA even when it was estimated exclusively from saccade‐contaminated epochs corrected with ICA. Previous work by Kang and Woodman (2014) and Mössing et al. (2024) reported that saccades during the retention interval—after the memory array had disappeared—did not affect CDA estimation. Our results are consistent with these findings and extend them, suggesting that saccades occurring during memory array presentation also do not distort the CDA if corrected with ICA. In contrast, when no correction is applied, residual saccadic noise that is not removed by epoch rejection can bias CDA amplitude estimation. This issue emerged only in the 500 ms array exposure condition, suggesting that for shorter durations, uncorrected saccades have less influence on the CDA when amplitude is measured as the mean within a predefined time window.

Not only was the CDA memory‐load effect preserved when participants executed saccades and ICA correction was applied, but CDA data quality actually improved (i.e., lower SME with ICA than with epoch rejection). This improvement was primarily due to the higher number of epochs retained after ICA correction. Thus, if the goal is to obtain CDA data quality comparable to standard experiments, applying ICA to correct lateral saccades could allow researchers to reduce the number of trials per participant.

Our control analyses further indicated that no confounding variables related to saccades remained in the CDA time window after ICA correction, since ICA successfully removed saccade‐related activity from both anterior and posterior electrode sites. Nevertheless, the SCN results leave an open question as to whether additional spurious cognitive processes might still contribute (see below). This shows that the CDA memory‐load effect observed after ICA correction genuinely reflects memory maintenance rather than residual saccadic activity. At the same time, comparisons between CDAs derived from uncorrected and ICA‐corrected contaminated epochs showed that the memory‐load effect can still be detected even without correction. This challenges the necessity of fine‐grained ocular artifact correction—except in cases where blinks occur during memory array presentation—if the sole aim is to demonstrate the presence of a memory‐load effect. However, when the goal is to obtain reliable CDA amplitude estimates or to investigate more detailed properties—such as the set size at which the CDA reaches its plateau—residual saccadic activity could artificially increase CDA magnitude as well as the plateau.

As predicted, differences between eye‐control conditions and memory array exposure times emerged when analyzing CDA onset and offset latencies. Specifically, CDA onset occurred earlier in the lateral saccade condition than in the central fixation condition. As hypothesized by us and others (Brisson and Jolicœur 2007; Drisdelle et al. 2017), this onset delay in the fixation condition may reflect the additional cognitive demand of maintaining fixation, which could affect attentional allocation timing. However, our SCN results suggest an alternative explanation. Consistent with Drisdelle et al. (2017), we observed the SCN component only when participants executed saccades, and importantly, it persisted after ICA correction. The presence of this component may explain the earlier CDA onset in the saccade condition.

Our data do not allow us to definitively identify the underlying cause of this earlier CDA onset, and a tailored study would be required to disentangle these possibilities. In particular, the memory‐load effect observed in the SCN could suggest that this component shares functional properties with the CDA and is sensitive to set size increases. Alternatively, it might reflect a delayed CDA onset in the fixation condition, or it could represent an overlapping process that temporally and spatially coincides with the CDA, producing what appears to be a memory‐load effect in an earlier time window. Given these uncertainties, we refrain from drawing strong functional conclusions about the SCN, beyond noting its potential contribution to earlier contralateral activity in the saccade condition.

As predicted, CDA offset latency was also affected: in the 500 ms exposure condition, the CDA ended earlier in the saccade condition than in the fixation condition. We hypothesize—and our results partially support—that this earlier offset may result from the extended presence of stimuli on the monitor, which provided a landing point for the saccade. Since the average saccade onset latency is 180–220 ms (Mayfrank et al. 1986)—a range also observed in our study—stimuli presented for only 100 ms typically vanish before the saccade begins, leaving the CDA largely unaffected. In contrast, with 500 ms exposures, participants had a visible target upon saccade completion, leading to bilateral representations once both foveae landed on the stimuli. This bilateral representation produced evenly distributed neural activity across hemispheres, eliminating the lateralized difference that defines the CDA. Consequently, the anticipated CDA offset disrupts its sustained nature and can distort mean amplitude estimates if the entire retention interval is analyzed. This finding extends Mossing and colleagues (Mössing et al. 2024), who investigated a process called remapping (Brincat et al. 2021; Golomb and Kanwisher 2012) and found that saccades during the retention interval did not reverse CDA polarity. This suggested that object representations remain anchored to their original hemifield, supporting a spatiotopic rather than a retinotopic code. In contrast, we observed that saccades during stimulus presentation resulted in initially lateralized stimuli being recoded bilaterally. Importantly, for a process to qualify as remapping, the saccade must occur after stimulus offset. Thus, our findings do not indicate remapping but rather suggest retinotopic recoding, where the same stimuli are encoded twice from different retinal positions. We note that this interpretation is inferred from the timing pattern, and a more direct test of retinotopic recoding would require an eye‐tracking setup specifically designed to dissociate these alternatives.

Interestingly, accuracy analyses revealed improved performance in the saccade condition compared to fixation, regardless of exposure time. The improvement in the 500 ms condition was expected due to more natural viewing conditions (longer encoding time and binocular vision after the saccade). However, the improvement in the 100 ms condition was unexpected. One possible explanation is that maintaining central fixation imposed an additional cognitive/attentional load, reducing performance regardless of exposure time. Another explanation relates to saccadic suppression. Previous studies (Brooks et al. 1981; Chekaluk and Llewellyn 1994; Gilchrist 2011; Ross et al. 2001) showed that saccades produce retinal image smearing, which is counteracted by saccadic suppression—a mechanism that reduces visual sensitivity to prevent motion perception. This mechanism can lead to backward and forward masking, impairing visual reporting from fixation just before and during a saccade. However, saccadic suppression predominantly affects rapid, low‐frequency luminance modulation (Ross et al. 2001), primarily suppressing the magnocellular pathway (motion detection) while sparing or even slightly enhancing the parvocellular pathway (color and high‐resolution form vision). Since our task required memorizing color, the reliance on parvocellular processing may have allowed participants to bypass suppression effects, potentially improving performance.

In conclusion, consistent with Drisdelle et al. (2017), our findings suggest that experimenters may adopt a more tolerant approach toward participants' lateral saccades in memory–probe tasks designed to measure the CDA, when stimulus exposure duration is less than the time of a saccade (~200 ms in adults) and ocular artifacts are corrected using ICA. A limitation of our study, however, is the absence of eye tracking, which would have allowed more precise characterization of oculomotor behavior. Instead, we relied on EOG data to estimate saccade onset, direction, and landing, which we consider sufficient for evaluating the impact of eye movements on CDA estimation across correction methods. Nevertheless, replication with eye‐tracking measures would strengthen and refine these conclusions.

If confirmed, these results would be particularly relevant, as they indicate that saccades—especially in response to briefly presented colored stimuli (< 200 ms)—do not necessarily disrupt the intended hemispheric lateralization. Future studies are needed to test whether these findings generalize to tasks involving other stimulus features. Importantly, however, we caution against applying ICA indiscriminately without considering experimental requirements. In long‐exposure conditions (> 200 ms), saccades result in early CDA offset and can bias average CDA amplitude across the entire retention interval, leading to an underestimation of memory load effects. For such conditions, if the whole retention interval is not of interest, or the group under study is more prone to eye movements (such as children or older adults), researchers may allow participants to saccade and analyze only the early part of the CDA (e.g., 300–650 ms), where we also observed a reliable memory‐load effect. If, however, the research goal is to examine CDA dynamics throughout the retention interval, shorter exposure times should be used when applying ICA—or epoch rejection should be preferred for longer durations.

## Author Contributions

**Alberto Petrin:** conceptualization, data curation, formal analysis, investigation, methodology, visualization, writing – original draft, writing – review and editing. **Sabrina Brigadoi:** conceptualization, data curation, methodology, visualization, writing – original draft, writing – review and editing. **Mattia Doro:** methodology. **Paola Sessa:** writing – original draft. **Roberto Dell'Acqua:** conceptualization, investigation, methodology, writing – original draft, writing – review and editing, project administration.

## Funding

The authors have nothing to report.

## Conflicts of Interest

The authors declare no conflicts of interest.

## Data Availability

The data that support the findings of this study are openly available in OSF (Open Science Framework) at https://osf.io/w5jqk/.
